# Sensory testing and topical capsaicin can characterize patients with rheumatoid arthritis

**DOI:** 10.1007/s10067-022-06185-0

**Published:** 2022-04-30

**Authors:** Bjoern Anders, Malte Anders, Matthias Kreuzer, Sebastian Zinn, Lukas Fricker, Christoph Maier, Miriam Wolters, Michaela Köhm, Frank Behrens, Carmen Walter

**Affiliations:** 1 Fraunhofer Institute for Translational Medicine and Pharmacology ITMP, Theodor-Stern-Kai 7, 60596 Frankfurt am Main, Germany; 2 Department of Anesthesiology and Intensive Care, Rechts Der Isar Hospital, Technical University of Munich School of Medicine, Ismaninger Str. 22, 81675 Munich, Germany; 3 Department of Anesthesiology, Intensive-Care Medicine and Pain Therapy, University Hospital Frankfurt, Goethe University Frankfurt Am Main, Theodor-Stern-Kai 7, 60590 Frankfurt, Germany; 4 University Hospital of Pediatrics and Adolescent Medicine, Ruhr-University Bochum, Bochum, Germany; 5 Department of Rheumatology, Goethe University Frankfurt Am Main, Theodor-Stern-Kai 7, 60590 Frankfurt, Germany

**Keywords:** Capsaicin, CHEPs, Rheumatoid arthritis

## Abstract

**Background and objectives:**

Our study aimed at examining the long-time inflammatory effects of rheumatoid arthritis (RA) as chronic immune-mediated disease on pain sensation and neuropathy development compared to healthy subjects (HS).

**Methods:**

We used the quantitative sensory testing (QST) protocol of the German Research Network on Neuropathic Pain and Electroencephalography (EEG)–based contact heat evoked potentials (CHEPs) before and after topical capsaicin application. We recruited 16 RA patients in remission or low disease activity state (mean age: 59.38 years [± 10.18]) and 16 healthy subjects (mean age: 56.69 years [± 8.92]).

**Results:**

The application of capsaicin cream on the thigh provoked a stronger effect in HS for both mechanical and heat pain thresholds (MPT and HPT, resp.), according to the area under the receiver operation characteristic (AUROC) (HS: HPT: 0.8965, MPT: 0.7402; RA: HPT: 0.7012, MPT: 0.6113). We observed contrary effects regarding changes in CHEPs (HS: g*max =  − 0.65; RA patients: g*max = 0.72).

**Conclusion:**

As the overall effect of topical capsaicin application was higher in HS for QST, we suggest the existence of a sensitization of TRPV1 channels in RA patients caused by long-time chronical inflammation, despite a lack of clinical signs of inflammation due to adequate treatment. The effect in CHEPs probably uncovers neuropathic symptoms. The effect of topical capsaicin on HPTs and CHEPs can act as a marker for the extent of sensitization and the development of neuropathic symptoms. Further studies are needed to prove if our proposed method can act as a marker for the success of anti-inflammatory treatment.

**Table:** 

**Key Points** • *The effect of topical capsaicin may represent the extent of TRPV1 sensitization in rheumatoid arthritis.* • *The effect of topical capsaicin on the amplitude level of CHEPs can unmask neuropathic symptoms.* • *The effect of topical capsaicin on CHEPs and HPTs can show the long-term consequences and the treatment success of RA patients in remission.*

## Introduction

Rheumatoid arthritis (RA) is an immune-mediated joint disease that can severely reduce function and quality of life. RA patients report pain as one of the most important factors for those negative effects [1], which serves as a substitute marker for impairment and is often associated with disease activity [2]. Nevertheless, RA patients show disease-associated pain problems even in the absence of inflammatory signs or low disease activity scores [3]. Disease-modifying antirheumatic drugs (DMARDs) usually represent the basic treatment for RA, while nonsteroidal anti-inflammatory drugs (NSAIDs) and glucocorticoids (GCs) are used for symptomatic treatment [4, 5]. Further options in case the basic therapy is insufficient are topical capsaicin, weak opioids, and treatments such as joint infiltrations or surgical management. However, a significant number of patients continue to experience pain despite adapted treatment [6]. Recent survey-based research emphasized that certain groups of RA patients even suffer from a neuropathic pain component, which would require treatment adjustment according to different treatment strategies than immunosuppressive drugs [7].

One method to detect sensory disorders and neuropathic pain is quantitative sensory testing (QST), which can provide valuable information about the underlying pathophysiological mechanisms of pain [8, 9]. Moreover, QST may allow predictions and reflections of treatment responses [10]. In previous studies with RA patients, only single elements of QST have been applied; e.g., the RA disease resulted in higher baseline pain levels in those patients who reported lower pressure pain thresholds (PPTs) [11]. While QST is a well-researched method for the detection and characterization of sensory disorders, it is fully dependent on the subjective input from the participants. Hence, recent research also focused on electroencephalography (EEG)–based contact heat evoked potentials (CHEPs), which is an established objective procedure to assess the function of the Aδ and C fibers [12]. It relies on EEG data, which is not dependent on the subjective clinical examination as compared to QST [13]. The readout as extracted from the EEG can serve as an indirect marker of the function of the nociceptive system [14, 15]. One possible use is the evaluation of the effects of analgesics on the objective response in the EEG after standardized noxious stimulation [16].

With our study, we intend to characterize the pain profile of well-treated RA patients using QST and CHEPs. To our knowledge, CHEPs have not been used to study the pain profile of patients with RA. We further analyze the sensitizing effects of topically applied capsaicin on QST and CHEPS recordings. We aim at identifying parameters to track the treatment response of RA patients and quantify the neuropathy development which may be caused by chronic inflammation and pain. We further aim at determining if either QST, CHEPs, or both are a suitable method to develop approaches for the stratified treatment of pain in RA patients.

## Materials and methods

### Study population

We included 16 patients (see Table 1) with a diagnosis of rheumatoid arthritis with low disease activity or in remission according to DAS28 < 3.2 (RA group, 8 male and 8 female, mean age: 59.38 years [± 10.18], PainDETECT score: 6.75 [± 5.57]) and 16 healthy subjects (HS group, 8 male and 8 female, mean age: 56.68 years [± 8.93]). Both groups were asked to refrain from taking pain medication of any kind for 5 days prior to the study visit. We excluded patients with a PainDETECT score over 18 [17], who currently abused alcohol or drugs (evaluated via verbal anamnesis), who took antidepressants, or who suffered from any other chronic pain or diagnosed neuropathic diseases. All subjects had a BMI < 30 kg/m^2^ and were of Caucasian ethnicity.

**Table 1:** Characteristics of subjects

Characteristics	% or mean [SD]	
	HS	RA
Age	56.69 years [± 8.92]	59.38 years [± 10.18]
Male/Female	50%/50%	50%/50%
Caucasian	100%	100%
BMI [kg/m^2^]	23.7 [± 3.67]	25.12 [± 3.42]
PainDETECT score		6.75 [± 5.57]
Treatment		
*No specific treatment*		12.50%
*MTX only*		12.50%
*TNF-inhibitor only*		25.00%
*MTX* + *TNF-inhibitor*		43.75%
*JAK-inhibitor*		6.25%
Disease duration		
*1–3 years*		6.25%
*3–5 years*		12.50%
*5–10 years*		56.25%
*10* + *years*		25.00%
CRP [mg/l]		3.26 [± 3.58]
SJC28 (swollen joint count)		0.31 [± 0.68]
TJC28 (tender joint count)		0.75 [± 1.35]
DAS28 (CRP-3)		1.68 [± 0.83]

Patients were only included if their RA treatments were unchanged for at least 3 months prior to the study, due to a stable disease state (remission or low disease activity with a DAS28 < 3.2). The subjects had to confirm to be mentally and physically able to manage the study procedure of about 4 h. We recruited the patients in the RA group from the Division of Rheumatology of the University Hospital of Frankfurt am Main (Germany). Both HS and RA groups underwent a training visit for about 30 min to assure they could consistently evaluate and tolerate both QST and CHEPs procedures.

### QST 

QST is a standardized protocol of the German Research Network on Neuropathic Pain (DFNS) using well-established tests for nearly all aspects of somatosensation by thermal and mechanical testing procedures. It consists of seven tests measuring 13 parameters [9, 18].

We performed three sets of QST measurements on the dominant body side: the first on the front of the thigh and the second on the back of the hand. Before the third measurement, we applied 200 mg of capsaicin cream (concentration: 0.2%) with an exposure time of 20 min and then measured a full set of QST on the front of the same thigh as in set one.

#### Thermal detection and thermal pain thresholds

The subject’s thermal sensation was determined using a TSA 2001-II (MEDOC, Israel) thermal sensory testing device. We determined the cold detection threshold (CDT) and warm detection threshold (WDT) by asking the subject to press a trigger button if they experienced any cold or warm sensation. The number of paradoxical heat sensations (PHS) was determined during the thermal sensory limen procedure (TSL; the difference limen for alternating cold and warm stimuli). The subjects had to press the trigger if they experienced any cold or warm sensation. Afterwards, we asked them to describe the sensation as cold or warm. We determined the cold pain threshold (CPT) and heat pain threshold (HPT) by asking the subjects to press the trigger if they experienced a painful component in addition to sensation of cold or heat. The mean threshold temperature of three consecutive measurements was calculated. Cutoff temperatures were 0 and 50 °C. We set the baseline temperature to 32 °C (center of neutral range) and the rectangular contact area of the thermode was 7.84 cm^2^ [9, 18].

#### Mechanical detection threshold (MDT)

We measured the mechanical detection threshold (MDT) to assess the Aβ fiber function with a standardized set of von Frey filaments (Marstock, Germany) of forces between 0.25 and 256 mN. The contact area was of uniform size and shape (round, 0.5 mm diameter). The mean of five series defined the threshold of ascending and descending stimulus intensities [9, 18].

#### Mechanical pain threshold (MPT)

The mechanical pain threshold (MPT) was determined to assess the Aδ fiber function with respect to mechanical allodynia. We used a set of PinPrick stimulators (MRC Systems, Germany) with a flat contact area with a diameter of 0.25 mm and forces between 8 and 512 mN. The threshold was calculated as the geometric mean of five series of ascending and descending stimulus intensities [9, 18].

#### Mechanical pain sensitivity (MPS)/dynamic mechanical allodynia (DMA)

Using PinPricks, we further tested mechanical pain sensitivity (MPS). To obtain a stimulus–response function, seven pinprick stimuli were applied in a balanced order with five repetitions for each PinPrick. We asked the subjects to give pain ratings for each stimulus on a 0–100 numerical rating scale (NRS) for 0 = no pain and 100 = most intense pain imaginable. Applying single strokes of a set of three light tactile stimulators (a cotton wisp, a cotton wool tip, and a standardized brush; five times each) of approximately 1–2 cm in length over the skin, we determined stimulus–response functions for dynamic mechanical allodynia (DMA). Subjects also rated pinprick stimuli on hand/thigh with a 0–100 NRS for each stimulus [9, 18].

#### Wind-up ratio (WUR)

At first, subjects rated a single 256 mN PinPrick stimulus. Afterwards, we applied a series of 10 stimuli with a frequency of 1/s. The subjects rated pain intensity on a scale from 1 to 100 after we applied all 10 stimuli. The procedure was repeated five times [9, 18].

#### Vibration detection threshold (VDT)

A Rydel–Seiffer tuning fork (64 Hz, 8/8 scale) placed over a bony prominence of the middle finger joint/knee for three times determined the vibration detection threshold, mediated by Aß fibers [9, 18].

#### Pressure pain threshold (PPT)

Three sets of slowly increasing stimuli (50 kPa/s) using a pressure gauge device (Jtech Medical, Midvale, USA) with a probe area of 1 cm^2^ determined the pressure pain threshold (PPT). PPT makes it possible to assess the pain sensitivity of the deep muscles, probably mediated by the muscles Aδ and C fibers [9, 18].

### Topical capsaicin

We applied 200 mg of capsaicin cream (capsaicin 0.2% incorporated in Basiscreme DAC) to the front of the thigh, followed by an exposure time of 20 min. The QST measurements were performed in this area for a third time and 200 mg of capsaicin cream was applied to the non-dominant forearm area as well, followed by an exposure time of 20 min. We then placed the thermode for CHEP measurements in this area and performed measurements using a stimulation temperature of 54 °C.

### CHEPs

Each study took place with the subject sitting in a quiet room. We equipped each subject with an EEG cap (Guger Technologies, Austria), which incorporated 21 active EEG electrodes according to the 10–20 system, attached to a g.Tec g.HIamp multichannel amplifier. We measured the location of the Cz electrode, which was placed midway between the nasion (most anterior point of the frontonasal suture) and inion (most prominent point of the occipital bone) and midway between both tragi. We used active EEG electrodes with a very low output impedance to minimize the influence of artifacts from the movement of the electrode cables [19]. We recorded the raw EEG using the g.Recorder software from g.Tec with a sample rate of 512 Hz.

Subjects underwent four series of CHEP stimuli with 48 °C, 51 °C, 54 °C, and 54 °C after topically applied capsaicin at the non-dominant volar forearm. Each series contained seven stimuli of each temperature with the inter stimulus interval set to 40 s. The subjects verbally rated every stimulus on a 0–100 NRS approximately 10 s after its appearance.

### EEG analysis

For data preprocessing, EEGLAB, a MATLAB-based (The MathWorks Inc., Natick, MA, USA) toolbox, was used [20]. We down-sampled the EEG to 256 Hz and applied a high-pass filter at 1 Hz and a low-pass filter at 42 Hz to eliminate 50 Hz line noise. We re-referenced the datasets to mathematically linked electrodes on the earlobes [21].

We cleaned the EEG data using Artifact Subspace Reconstruction (ASR) with a threshold of 20 standard deviations [22] and then epoched the data from − 1 to + 2 s around the stimulus onset.

### Statistical analysis

We carried out the statistical analysis using MATLAB. We logarithmized the QST data of CDT, WDT, PPT, MPT, MPS, DMA, WUR, and MDT and kept the rest of the data in the original format [23].

We confirmed the normal distribution of the QST and NRS_100_ data with the Kolmogorov–Smirnov test. We compared the data using a two-sample *t* test and calculated the area under the receiver operating characteristics (AUROC) for HPTs, MPTs, and CHEPs-NRS ratings with MATLAB’s toolbox “ROC Curve.” AUROC has been chosen as this method has proven to be able to differentiate between two conditions, i.e., in our case between RA patients and HS [24].

We calculated the *p* values for CHEPs using the Mann–Whitney *U* test and corrected the obtained *p* values for multiple comparisons with Bonferroni correction. The significance level was set to *p* < 0.05. Furthermore, we calculated Hedges’ *g* effect size [g*] at every data point [25]. As a rule of the thumb, absolute effect sizes above 0.5 present a “medium” effect [26]. Hence, we only considered g* >|0.5| as relevant.

## Results

### Comparison of QST values of RA patients versus healthy subjects (see Table 2)

**Fig. 1:**
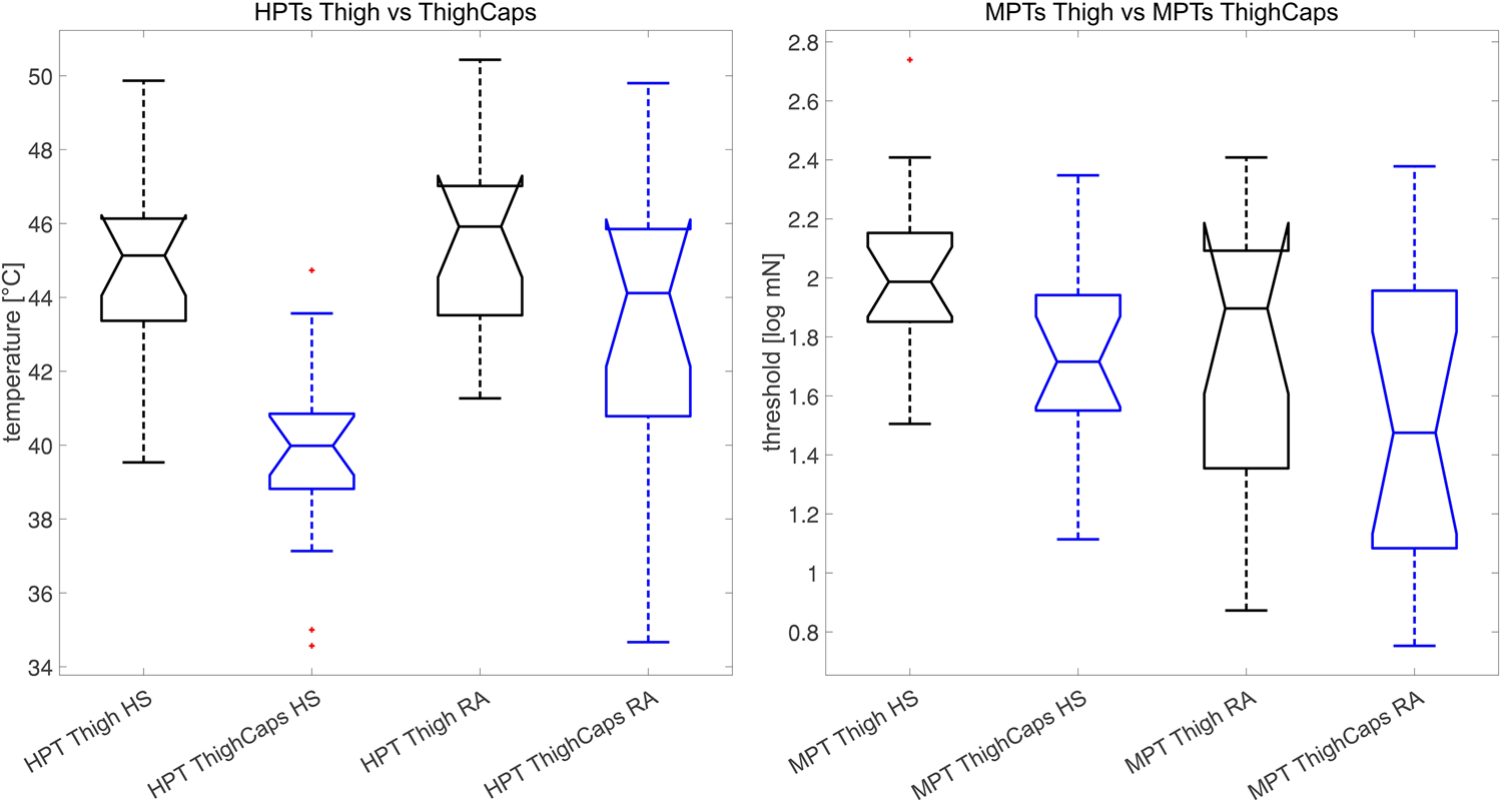
Boxplot of HPTs (left) and MPTs (right) before vs after capsaicin application in HS and RA patients (red cross = outlier)

**Fig. 2:**
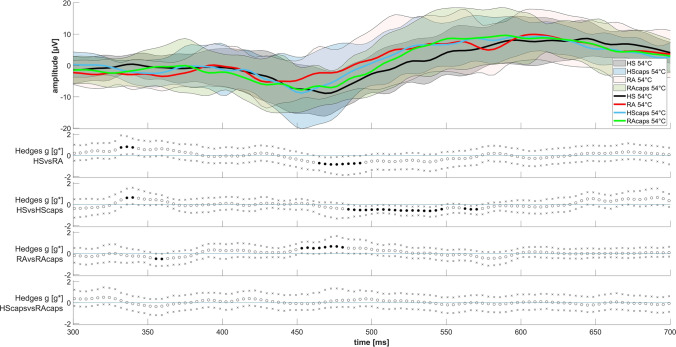
Average event-related potential (ERP) waveforms from 300 to 700 ms following the 54 °C stimulus before and after the application of capsaicin cream. The lines indicate the average amplitude at Cz electrode location for all subjects of each group. Comparison between the groups was carried out by plotting the Hedges *g* effect size [g*]. Dots are of white color if *p* > 0.05 and of black color if *p* < 0.05

We observed only significant differences for PPT values on the hand (*p* = 0.0264). While HPTs (as also all other parameters were within the normal range) on the hand (*p* = 0.8912) and on the thigh (*p* = 0.2595) did not yield a significant difference, the application of capsaicin cream on the thigh led to a significant difference between both groups for WDTs (*p* = 0.0282) and HPTs (*p* = 0.0277). We compared the effect of capsaicin in both groups on the HPTs (see Fig. 1; HS: *p* < 0.0001; RA: *p* = 0.0316) and on the MPTs (see Fig. 2; HS: *p* = 0.0186; RA: *p* = 0.2136) between before and after the application.

**Table 2:** Means/SDs of QST values and *p* values/CIs of HS versus RA patients. We show the 95% CI for the difference between means of HS and RA

QST parameters		Healthy subjects		RA patients			
		Mean	SD	Mean	SD	*p*	HS-RA [95% CI]
Hand							
CDT	Log(ΔT)	0.31	0.21	0.40	0.25	0.2718	− 0.09 [− 0.25; 0.07]
WDT	Log(ΔT)	0.59	0.34	0.66	0.24	0.5504	− 0.07 [− 0.27; 0.15]
TSL	Log(T)	0.81	0.26	0.90	0.24	0.3227	− 0.09 [− 0.27; 0.09]
CPT	°C	17.15	6.24	15.74	7.16	0.5571	1.41 [− 3.44; 6.26]
HPT	°C	44.69	2.82	44.82	2.37	0.8912	− 0.13 [− 2.01; 1.76]
MDT	Log(mN)	0.51	0.62	0.18	0.56	0.1215	0.33 [− 0.09; 0.76]
MPT	Log(mN)	1.79	0.48	2.04	0.54	0.1797	− 0.25 [− 0.61; 0.12]
MPS	Log(NRS_100_)	− 0.40	0.42	− 0.42	0.30	0.8973	0.02 [− 0.25; 0.28]
WUR		0.47	0.26	0.48	0.26	0.9314	− 0.01 [− 0.20; 0.19]
PPT*	**Log(kPa)**	**2.71**	**0.14**	**2.59**	**0.14**	**0.0264**	**0.12 [0.01;** − **0.21]**
Thigh							
CDT	Log(ΔT)	0.57	0.27	0.51	0.19	0.4179	0.06 [− 0.09; 0.21]
WDT*	**Log(ΔT)**	**0.40**	**0.09**	**0.58**	**0.19**	**0.0029**	− **0.18 [**− **0.29;** − **0.06]**
TSL*	**Log(T)**	**0.85**	**0.13**	**0.96**	**0.18**	**0.0495**	− **0.11 [**− **0.23; 0.00]**
CPT	°C	13.60	9.44	13.55	8.05	0.9872	0.05 [− 6.29; 6.39]
HPT	°C	44.60	2.69	45.69	2.68	0.2595	− 1.09 [− 3.03; 0.85]
MDT	Log(mN)	0.52	0.43	0.49	0.59	0.8808	0.03 [− 0.35; 0.40]
MPT	Log(mN)	2.02	0.31	1.77	0.43	0.0718	0.25 [− 0.02; 0.52]
MPS*	**Log(NRS**_**100**_**)**	− **0.46**	**0.29**	− **0.16**	**0.44**	**0.0258**	− **0.30 [**− **0.57; 0.04]**
WUR		0.47	0.50	0.48	0.24	0.9298	− 0.01 [− 0.30; 0.27]
PPT	Log(kPa)	2.82	0.13	2.73	0.15	0.0839	0.09 [− 0.01; 0.19]
Thigh + capsaicin cream							
CDT	Log(ΔT)	0.69	0.21	0.57	0.29	0.1857	0.12 [− 0.06; 0.30]
WDT*	**Log(ΔT)**	**0.45**	**0.13**	**0.57**	**0.15**	**0.0282**	**0.30 [**− **0.22;** − **0.01]**
TSL	Log(T)	0.95	0.18	0.97	0.15	0.7585	− 0.02 [− 0.14; 0.10]
CPT	°C	11.55	8.18	7.73	9.63	0.2351	3.82 [− 2.62; 10.28]
HPT*	**°C**	**39.81**	**2.71**	**42.79**	**4.39**	**0.0277**	− **2.98 [**− **5.62;** − **0.35]**
MDT	Log(mN)	0.63	0.42	0.56	0.41	0.6458	0.07 [− 0.23; − 0.36]
MPT	Log(mN)	1.73	0.34	1.56	0.51	0.2695	0.17 [− 0.14; 0.49]
MPS	Log(NRS_100_)	− 0.23	0.33	− 0.07	0.44	0.2691	− 0.30 [− 0.44; 0.13]
WUR		0.62	0.41	0.45	0.17	0.1364	0.17 [− 0.06; 0.40]
PPT	Log(kPa)	2.73	0.24	2.74	0.13	0.8441	− 0.01 [− 0.15; 0.13]

Significant differences are shown in bold

### AUROC analysis of HPT values

Due to the incoherent effect of capsaicin cream on the HPT and MPT, we also calculated AUROC (see Table 3). Those values demonstrated a more robust separation effect in HS (HPT: 0.88, MPT: 0.70) compared to RA patients (HPT: 0.73, MPT: 0.63) before and after the application of capsaicin cream.

**Table 3:** AUROC analysis of HTPs and MPTs (thigh) before and after the application of capsaicin cream

Compared values	AUROC value	Sensitivity	Specificity
HPTs HS vs RA patients	0.61	0.69	0.63
HPTs after capsaicin application HS vs RA patients	**0.76**	**0.75**	**0.81**
HPTs before vs after capsaicin application in HS	**0.90**	**0.81**	**0.88**
HPTs before vs after capsaicin application in RA patients	**0.70**	**0.69**	**0.69**
MPTs HS vs RA patients	0.67	0.69	0.69
MPTs after capsaicin application HS vs RA patients	0.59	0.81	0.50
MPTs before vs after capsaicin application in HS	**0.74**	**0.75**	**0.69**
MPTs before vs after capsaicin application in RA patients	**0.61**	**0.69**	**0.56**

Relevant separations are shown in bold

### CHEPs

We did not observe any significant differences or effect sizes above 0.5 or below − 0.5 for the temperatures of 48 °C and 51 °C respectively between both groups. Hence, we only plotted the CHEPS for 54 °C stimulation temperature in Fig. 2. Regarding the magnitude of the n-amplitude at 54 °C stimulation temperature, HS and RA patients showed a significant difference with a maximum effect size of g*_max_ =  − 0.95 at 469 ms. After the application of capsaicin cream, HS experienced an effect with a negative g*_max_ =  − 0.65 at 535 ms and RA patients with a positive g*_max_ = 0.72 at 473 ms. We observed no significant difference comparing both groups after the application of capsaicin cream (see Fig. 2). NRS ratings (see Table 4) did not yield differences before and after capsaicin application and the AUROC analysis did not show any relevant separation (HS: AUROC = 0.57, *p* = 0.1646; RA patients: AUROC = 0.50, *p* = 0.4486).

**Table 4:** NRS ratings following the 54 °C stimulus before and after the application of capsaicin cream. We show the 95% CI for the difference between means of HS and RA

NRS [0–100] ratings											
HS						RA patients					
	Mean [NRS_100_]	SD	***p***	95% CI (HS–RA)	AUROC		Mean [NRS_100_]	SD	***p***	95% CI (HS-RA)	AUROC
54 °C	27.85	22.64	0.1646	− 10.06, 1.72	0.57	54 °C	26.48	21.81	0.4486	− 8.99, 3.99	0.50
54 °C + Caps	32.02	22.11				54 °C + Caps	29.98	27.19			

## Discussion

This study provides an extensive QST data set combined with measurements of CHEPs in RA patients in remission or low disease activity and a control group of healthy subjects.

PPT measurements on the hand yielded a significant difference between RA and HS, which corresponds to the results of previous studies [11, 27]. However, in contrast to past studies, the included RA patients did not declare lower HPTs [28]. Comparing the AUROC values for the difference in HPT values after the application of capsaicin cream underlines a stronger separation in HS (AUROC ~ 0.90, *p* < 0.0001) vs RA patients (AUROC ~ 0.70, *p* ~ 0.03), underlining possibly a more crucial impact of the compound in HS. The underlying mechanisms for these observations are not yet clear. We assume an overexpression or sensitization of TRPV1 receptors in RA patients [29]. Regarding our results, HS experience a stronger sensitization effect than RA patients after the application of capsaicin cream for both HPTs and MPTs, according to the AUROC values.

Although the capsaicin-induced area of hyperalgesia is larger in RA patients with active disease [30], potentially caused by overexpressed or sensitized TRPV1 receptors, we observed a clearer separation effect for the MPT change in HS (AUROC ~ 0.74, *p* ~ 0.02) than in RA (AUROC ~ 0.61, *p* ~ 0.21) after the application of capsaicin cream. This overexpression is shown in more recent research [29]. The study did not report any NRS ratings in addition to the area of hyperalgesia on that we were able to show a clearer effect in HS.

Other agonists for TRPV1 besides capsaicin are endogenous components like substance P and calcitonin gene–related peptide, two of the main neuropeptides involved in the development of inflammation. Both peptides increase the TRPV1 expression in RA synoviocytes [29]. TRPV1 deletion may block the pro-inflammatory function, thereby reducing synovial inflammation. Among other procedures, deletion of gene segments responsible for the expression of TRPV1 prevented the progression of RA and the establishment of hyperalgesia priming. This resulted in overall decreased disease activity and pain sensation during the chronic phase of RA. TRPV1-deficient mice with experimental RA showed decreased synovial inflammation, bone erosion, and cartilage damage in the joint in the early disease phase [31]. The expression of TRPV1 increased after inflammation whereas TRPV1 deletion inhibited synovial macrophages [31]. Different changes in the expression of TRPV1 are described. In inflammation models, there is an increase in TRPV1 expression at the RNA and protein level, whereas in models of neuropathic pain there is sometimes a reduction [32].

In our study, for both MPTs and HPTs, the sensitization effect of topical capsaicin is weaker in RA patients, compared to HS. This observation might rather result from sensitization processes due to RA-related inflammation than from difficulties to sensitize TRPV1 channels in RA patients, even in the absence of clinical inflammation signs. This context might reduce the potential for a sensitization effect of topical capsaicin to the same extent as in HS. The influence of inflammatory mediators on TRPV1 and nociception may also be different. Both pronociceptive and antinociceptive influences can be mediated by modification at TRPV1 [32].

Excessive activation of the TRPV1 channel, possibly through permanent activation by the inflammatory soup as nociceptive input with the associated Ca2 + influx, ultimately leads to desensitization of the nociceptor and the associated reduced pain sensitivity. It also means a loss of function, which may be irreversible [33]. This could contribute to the development of a neuropathic pain type with mechanical pain threshold changes [34]. The observed changes in PPT for deep mechanical pain in RA patients show such a difference compared to healthy subjects.

Previous studies utilized QST to identify subgroups of neuropathic pain with different etiologies and classify treatment responders [35]. Due to our small sample size, we did not perform a subgroup analysis. This study, however, shows that this is a possible approach for RA patients. Larger study populations might yield similar clusters, with topically applied capsaicin as a supplementary measure. A recent publication analyzed the relationship between RA, COVID-19, and the immune-neuroendocrine system and concludes that RA patients have an altered response of the immune-neuroendocrine system [36]. However, our data does not support any further conclusions regarding a possible effect of capsaicin on the immune-neuroendocrine system.

For CHEPs, regarding the maximum amplitudes, other studies showed a latency-shortening effect of topical capsaicin in healthy subjects [37]. We further revealed a maximum amplitude change for the n-wave after capsaicin application in RA patients to − 8.77 µV, whereas before the n-wave was unclear to detect. This change was almost undetectable in HS (− 8.77 to − 9.51 µV).

Our CHEP results suggest that pain that is assessed in RA patients in remission state can be characterized as neuropathic symptom with need for a different treatment strategy than change or intensification of immune-suppressive therapy. Comparing the event-related potentials (ERPs) of 54 °C stimuli between HS and RA patients, RA has a strong negative effect on the height of the n-amplitude (g*_max_ =  − 0.95), which corresponds to past research for neuropathic patients [38–40]. The application of capsaicin cream causes a positive impact on the height of the n-amplitude in RA patients (g*_max_ = 0.72). The negative impact in HS (g*_max_ =  − 0.65) could be deceiving, as it is most likely a shortened latency, and the maximum amplitude hardly changes. This effect corresponds to past research as well, where a latency-shortening effect of topical capsaicin in healthy subjects has already been shown [37]. After application of the capsaicin cream, no significant area of distinction can be shown between both groups, as the topical capsaicin could mask neuropathies of RA patients by sensitization. Interestingly, we cannot observe a similar sensitization effect on the n-amplitude in HS. For this, we propose that CHEP amplitudes at some point reached a kind of maximum, which was already triggered at 54 °C in HS. In RA patients, due to neuropathic symptoms, this is only possible through further sensitization, whereby no significant differentiation is possible after the application of capsaicin cream between both groups.

## Conclusion

We conclude that carrying out either QST or CHEPs alone might not be sufficient to show a possible (peripheral/central) pain sensitization of RA patients in remission to pain stimuli or any neuropathic component. We were able to show a diverging reaction to the capsaicin cream between those groups using both these methods. Our results are applicable to RA patients in remission or low disease activity with a stable treatment and a maximum NRS_10_ rating of RA pain of 3/10 before the study sessions. Thus, with the study setup described in this paper, TRPV1 sensitization and neuropathy development regarding the effect of capsaicin cream on their HPTs/MPTs and ERP images can be evaluated by comparing RA patients with a HS group.

Topical capsaicin application can be utilized to evaluate a possible neuropathy or sensitization/overexpression of TRPV1 channels in RA patients or similar inflammatory diseases. Applying CHEPs or HPT before and after capsaicin application, both methods were sufficient to show representable diverging effects already in a small study population. The comparison of different RA treatment approaches or disease durations and their effect on the described reaction to topical capsaicin is a possible application of these methods in future research. Combining QST and CHEPs before and after the application of topical capsaicin is thus advisable for study setups that compare inflammatory diseases with healthy control groups. The change in TRPV1 expression or the extent of a neuropathy development may show the ability of medications to suppress chronic inflammation and pain. The effect of topical capsaicin on HPTs or CHEPs might outline insufficient treatments of chronic inflammations in RA or the need for an additional treatment of neuropathic symptoms. Future studies need to prove if treatment changes can prevent or reduce the physiological effects of RA that are described in this manuscript.
